# A Novel Homozygous *GPAA1* Variant in a Patient with a Glycosylphosphatidylinositol Biosynthesis Defect

**DOI:** 10.3390/genes14071444

**Published:** 2023-07-14

**Authors:** Paolo Fontana, Alberto Budillon, Domenico Simeone, Francesca Del Vecchio Blanco, Martina Caiazza, Alessandra D’Amico, Fortunato Lonardo, Vincenzo Nigro, Giuseppe Limongelli, Gioacchino Scarano

**Affiliations:** 1 Medical Genetics Unit, P.O. Gaetano Rummo, A.O.R.N. San Pio, Via dell’Angelo, 1, 82100 Benevento, Italy; domenicosimeone@icloud.com (D.S.); fortunato.lonardo@aornsanpio.it (F.L.); gioac.scarano51@gmail.com (G.S.); 2 Department of Precision Medicine, University of Campania “Luigi Vanvitelli”, Via L. De Crecchio 7, 80138 Naples, Italy; alberto.budillon@hotmail.it (A.B.); francesca.delvecchioblanco@unicampania.it (F.D.V.B.); vincenzo.nigro@unicampania.it (V.N.); 3 Inherited and Rare Cardiovascular Disease Unit, Department of Translational Medical Sciences, University of Campania “Luigi Vanvitelli”, Via L. Bianchi, 80131 Naples, Italy; martina.caiazza@yahoo.it (M.C.); giuseppe.limongelli@unicampania.it (G.L.); 4 Department of Radiology, “Tortorella” Private Hospital, Via Nicola Aversano, 1, 84124 Salerno, Italy; damicoalex@tiscali.it; 5 Telethon Institute of Genetics and Medicine, Via Campi Flegrei 34, 80078 Pozzuoli, Italy; 6 Institute of Cardiovascular Sciences, University College of London and St. Bartholomew’s Hospital, London WC1E 6DD, UK

**Keywords:** *GPAA1*, cerebellar atrophy, hypotonia, transamidase complex

## Abstract

Glycosylphosphatidylinositol biosynthesis defect 15 is a rare autosomal recessive disorder due to biallelic loss of function of GPAA1. At the moment, less than twenty patients have been reported, usually compound heterozygous for GPAA1 variants. The main clinical features are intellectual disability, hypotonia, seizures, and cerebellar atrophy. We describe a 4-year-old male with a novel, homozygous variant. The patient presents with typical features, such as developmental delay, hypotonia, seizures, and atypical features, such as macrocephaly, preauricular, and cheek appendages. When he was 15 months, the cerebellum was normal. When he was 33 months old, after the molecular diagnosis, magnetic resonance imaging was repeated, showing cerebellar atrophy. This case extends the clinical spectrum of the GPAA1-related disorder and helps to delineate phenotypic differences with defects of other subunits of the transamidase complex.

## 1. Introduction

Congenital disorders of glycosylation (CDG) are a large group including more than 130 inherited metabolic diseases with multi-organ involvement caused by defects in genes that encode related enzymes in the oligosaccharide biosynthesis pathways [1]. The consequence is a disruption of the process of glycosylation, which is the attachment of sugar molecules to proteins and lipids. Glycosylation is one of the most significant and abundant post-translational modifications in mammalian cells. It mediates a wide range of biofunctions, including cell adhesion, cell communication, immune cell trafficking, and protein stability.

One subgroup of CDG is the disorders of glycosylphosphatidylinositol (GPI) biosynthesis. In disorders of GPI biosynthesis, there is a defect in the biosynthesis pathway of GPI, resulting in abnormal or insufficient GPI anchors. Individuals with disorders of GPI biosynthesis experience a wide range of symptoms, depending on the specific gene variants and the proteins affected. Some common features include neurologic abnormalities, intellectual disability, developmental delay, seizures, muscle weakness, and abnormal facial features. Several subtypes of disorders of GPI biosynthesis have been identified, each associated with variants in different genes involved in GPI anchor synthesis [2].

More than 100 human proteins located on the plasma membrane carry a GPI-anchor, composed of phosphatidylinositol and glycan chains, which mediate the binding to the cell membrane [3]. GPI-anchored proteins can be expressed both on the apical or basolateral surface of the membrane, can work as receptors, adhesion molecules, enzymes, and transporters, and have important roles in several cellular processes, including embryogenesis and tissue differentiation [4].

GPI-anchored proteins undergo a complex process of post-translational modifications in the endoplasmic reticulum. They are synthesized as pre-pro-proteins, and then they lose an N-terminal signal peptide, resulting in a pro-protein. Finally, the GPI transamidase complex mediates the last step of their processing by removing the C-terminal signal peptide and attaching, by an amide bond, a GPI-anchor [5]. GPI transamidase complex consists of five enzymes encoded by *PIGK*, *PIGS*, *PIGT*, *PIGU*, and *GPAA1*, respectively [6].

Although other GPI-biosynthesis defects are known, the first ten patients with a disorder associated with *GPAA1* variants were described only in 2017. This gene is located on chromosome 8 (8q24.3). The main clinical features of the patients are intellectual disability, hypotonia, both of them always reported, seizures and cerebellar atrophy, affecting more than an half of the patients. Microcephaly and osteopenia have been described, with a lower prevalence, in some of the affected individuals [6].

A significant clinical variability has been observed in several features, including the degree of intellectual disability (usually moderate to severe, but mild disability has been reported) and the pattern of the seizures. The facial phenotype is quite aspecific, although some features seem to be recurrent, such as a prominent forehead, a broad nose, and a tented upper lip [7].

*GPAA1*-related GPI biosynthesis defect recognizes an autosomal recessive mode of inheritance. Heterozygous variants have been supposed to determine a predisposition to vascular anomalies [8], but this association has not been confirmed by other authors.

Among the eighteen individuals up to now reported, including this patient, seven of them carry homozygous variants. This can arise from consanguineous marriages, the presence of genetic isolates, founder effect.

## 2. Clinical Report

**Figure 1:**
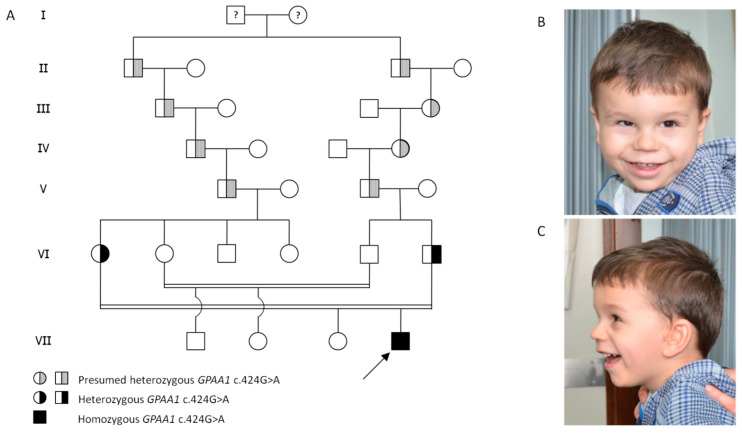
(**A**) Pedigree of the patient. Two great-great-great grandfathers of the child were brothers. One of them was probably heterozygous for the variant c.424G > A in the gene *GPAA1*. (**B**,**C**) Photographs of the patient at the age of 4. He shows strabismus, deep-set eyes, thin eyebrows, posteriorly rotated ears with preauricular tag, depressed nasal root with wide nasal tip, long and deep philtrum, and a thin and tented upper lip.

The patient is a 4-year-old male, born from the second pregnancy of parents with ninth-degree consanguinity (see Figure 1).

The mother had natural childbirth after 40 weeks of a normal pregnancy. Birth weight was 4060 g (1.71 SDS), length 50 cm (−0.3 SDS), head circumference 36.5 cm (1.62 SDS). At birth, the newborn presented with congenital muscular torticollis, treated with physiotherapy, bilateral preauricular appendages, and a left cheek appendage, removed in the first months of life. At three months, the baby was not able to control his head and showed hypotonia affecting the trunk and the neck.

At our first dysmorphologic assessment, the patient was 4 months old and presented with hypotonia, relative macrocephaly, mild frontal bossing, horizontal nystagmus, malar hypoplasia, left preauricular tag (right preauricular and left cheek tag had been already removed), depressed nasal root, bulbous nasal tip, anteverted nares, long philtrum. Heart ultrasound, abdominal ultrasound, spine X-ray, Auditory Brainstem Response, and blood routine tests, including alkaline phosphatase, were normal. SNP-microarray did not detect any chromosomal deletions nor duplications.

**Figure 2:**
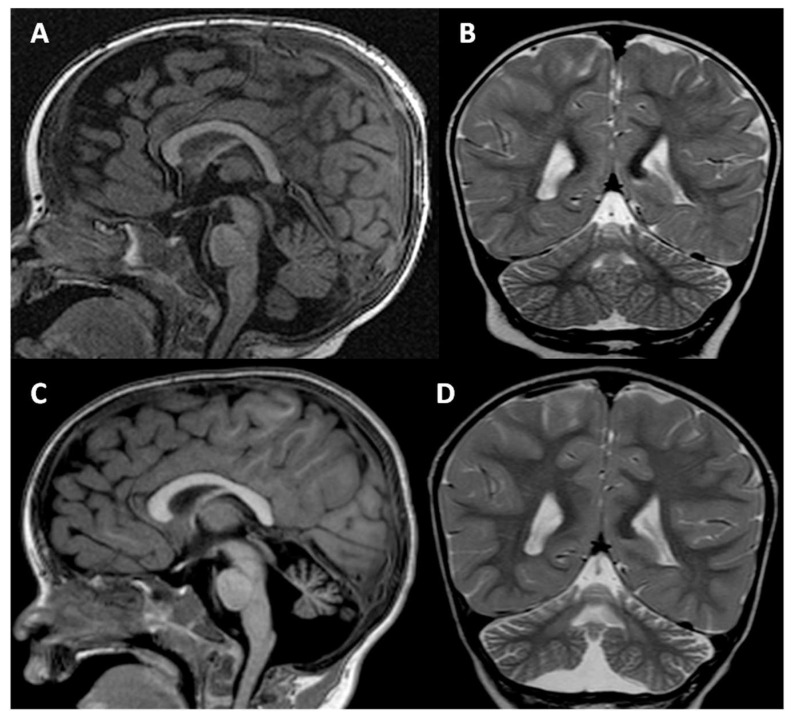
MRI of the brain was performed when the patient was 3 months (**A**,**B**) and 3 years old (**C**,**D**). Both midline sagittal T1-weighted and coronal T2-weighted images demonstrate a progressive reduction in cerebellar volume, especially of the vermis, with diffuse atrophy of the folia and secondary enlargement of the subtentorial CSF spaces. The *corpus callosum* is normal except for minimal hypoplasia of the rostrum (**A**,**C**). Note also the pathological progressive increased hyperintensity on T2-weighted images of the dentate nuclei (**B**,**D**).

When the patient was 31 months old, he developed seizures, which were treated and controlled with levetiracetam. Two MRI scans of the brain, which the patient underwent when he was 3 months and 3 years old, showed progressive atrophy of the whole cerebellum, but especially of the vermis, with increased pathological and symmetrical hyperintensity on T2-weighted images of the dentate nuclei. The *corpus callosum* was normal except for minimal hypoplasia of the rostrum. The brainstem remained normal in both MRIs. The second MRI showed the appearance in the deep left temporal white matter of an oval-shaped area, hyperintense on T2-weighted images, about 1 cm in size, without peripheral vasogenic edema an enhancement after gadolinium injection, currently under follow-up (Figure 2).

At our last evaluation, the patient was 4 years and 3 months old, his length was 97 cm (−1.75 SDS), his weight was 14 Kg (−2 SDS), and his head circumference was 51 cm (+0.3 SDS). He showed strabismus, depressed nasal root with wide nasal tip, long and deep philtrum, and tented upper lip (Figure 1). He was able to sit without support steadily and to stand for a few seconds, but he could not walk. The verbal language was absent. Eye contact was present. A bone density scan was performed and found to be normal.

## 3. Materials and Methods

Patient. The patient examined is of Caucasian origin. He comes from a small town in the province of Benevento (Campania, Italy) and is the son of consanguineous parents. His parents come from two small neighboring towns (1800 and 2200 inhabitants, respectively).

Informed consent and DNA samples. Informed consent was appropriately obtained for genetic investigations through next-generation sequencing (NGS) with an exome platform in a trio encompassing all the known genes.

DNA samples were obtained from fresh peripheral blood samples using the extraction kit FlexiGene DNA kit (Qiagen, Hilden, Germany, 2022). The genomic DNA was isolated from 1 mL of blood following the manufacturer’s recommendations. Genomic DNA was quantified with NanoDrop (IMPLEN, 2022).

Library preparation and sequencing. For library preparation, we followed the manufacturer’s instructions (SureSelectQXT Automated Target Enrichment for the Illumina Platform, Protocol Version B0, November 2015, Agilent Technologies, Santa Clara, CA, USA). The genomic DNA was enriched using the SureSelect Human All Exon v7 (Agilent Technologies, Santa Clara, CA, USA). The libraries were sequenced using the NovaSeq 6000 system performing paired-end runs covering at least 2 × 150 nt (Illumina Inc., San Diego, CA, USA). The generated sequences were analyzed using an in-house pipeline designed to automate the analysis workflow [9]. The average exome coverage of the target bases of at least 100×, with 90% of the bases covered by at least 40 reads.

Sanger sequencing validation. Following our workflow, the variant detected has also been validated through Sanger sequencing by adopting forward primer (TTCAGCCCCCTACCACAAAG) and reverse (CTTCAAGCCAAGCCTCAGTG).

## 4. Results

**Figure 3:**
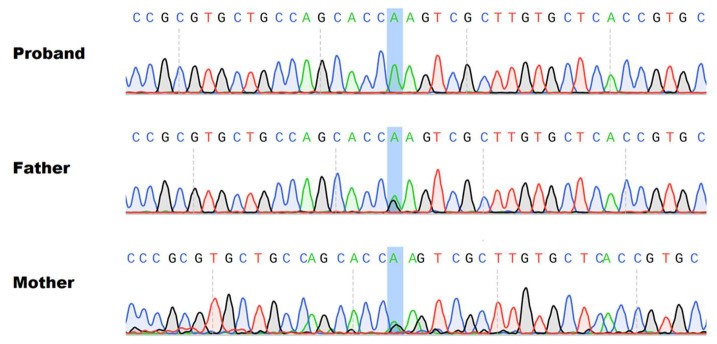
Following WES, Sanger sequencing provides the validation of the homozygous variant of the proband and the heterozygous state of both parents.

Exome analysis in a trio revealed a homozygous missense variant in the *GPAA1* gene (MIM 603048), NM_003801:c.424G > A (exon 4) producing p.Glu142Lys, and then confirmed by Sanger sequencing (Figure 3). As expected, both parents show the same heterozygous variant.

**Figure 4:**
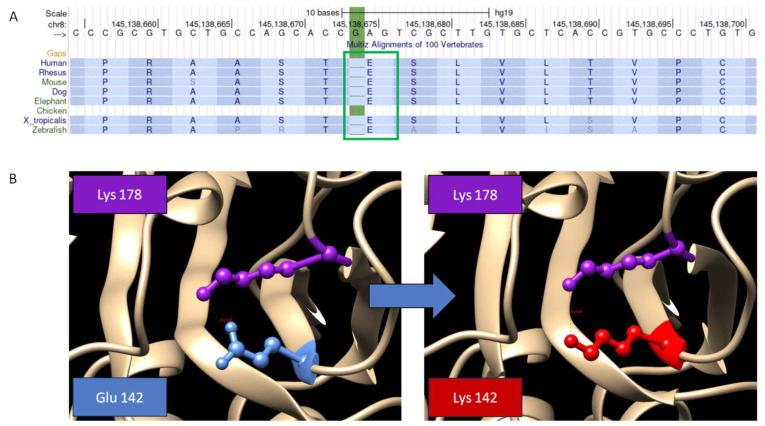
(**A**) Glutamic acid is a highly conserved amino acid across species suggesting its crucial role in the development of the protein. (**B**) 3D protein structure highlights the Glu to Lys amino acid change. Negatively-charged wilde-type Glu 142 may interact with positively-charged Lys 178, which is at a distance of 3.876 Å. Through a simulation, we could speculate that, when mutated, Lysine 142 charge may repel Lys 178 charge at a distance of 4.638 Å, altering the strand structure.

This nucleotide change was not present in the GnomAD database (https://gnomad.broadinstitute.org/) and had a CADD score of 26.8. The variant classification is Variant of Uncertain Significance, according to the ACMG criteria PP4, PP3, PM2, BP1, in detail: PP4: Patient’s phenotype or family history is highly specific for a disease with a single genetic etiology; PP3: MetaRNN = 0.982 is greater than 0.939 ⇒ strong pathogenic; PM2: Variant not found in gnomAD genomes, good gnomAD genomes coverage = 32.2; BP1: 41 out of 56 non-VUS missense variants in gene *GPAA1* are benign = 73.2% which is more than threshold of 33.1%. Even though it was classified as VUS according to the ACMG criteria, it was inferred to be deleterious according to many predictors, such as SIFT, Mutation Assessor, and PolyPhen 2.0. If compared with reference genomes of other species, the wild-type amino acid appears to be highly conserved, advising a key role in the protein architecture (Figure 4). The variants reside in a segment of amino acids that forms a repeating pattern in the GPAA1 protein, known as the lumenal repeat. Almost every ClinVar likely-pathogenic variants (PLPs) missense variant occurs within this domain. Introducing a different amino acid could potentially disrupt this repeating pattern and consequently impact any functional role associated with it.

Through interactive software for the visualization and the analysis of molecular structures (UCSF Chimera), we realized that the mutated Lys 142 electric interaction with the near Lys 178 residue may reshape the protein structure. The wild-type residue charge is negative, and the mutant residue charge is positive. Thus, while wild-type Glu 142 possibly attracts Lys 178, mutated Lys 142 is likely to repel it (Figure 4). Since the *GPAA1* gene is known to cause Glycosylphosphatidylinositol biosynthesis defect 15 (#617810), which matches with the patient phenotype, we could then consider the variant to be responsible for the condition.

## 5. Discussion

*GPAA1* defects determine a recessive disorder characterized by a complex neurological phenotype, including intellectual disability, dysarthria, nystagmus, spasticity, ataxic gait, hypotonia, seizures, and cerebellar atrophy.

Cerebellar hypoplasia and atrophy are features shared with other GPI-anchored protein defects, emphasizing the role of these proteins during cerebellar development. The genes coding for transamidase complex subunits follow a restricted pattern of expression in the early postnatal brain. Reduced activity of the complex does not grossly affect the structure of the brain, but it impairs cerebellum micro-organization and Purkinje cell dendritic arborization [10].

The neurological phenotype differs from other, more common, conditions, such as spinocerebellar ataxias, because of the very early onset and the absence of involvement of the brainstem.

Consistently with the organization of the glycophosphatidylinositol (GPI) transamidase complex into five subunits, variants in their respective five genes are associated with a common phenotype. The intellectual disability is usually severe, with poor or absent speech. Spastic tetraparesis, optic atrophy, cortical atrophy, and cortical blindness are frequent. Other neurological findings are recurrent, including cerebellar ataxia or dysmetria, hypotonia, strabismus, and nystagmus.

Nevertheless, peculiar phenotypical features have been related to specific genes. Patients with *PIGS* variants show a coarse facial appearance, with arched eyebrows, long noses, deep philtrums, broad tongues, and gingival hypertrophy [11]. Scoliosis and other skeletal findings are more frequent in patients with *PIGT* and *PIGU* variants [12,13]. Nephrocalcinosis and tooth abnormalities are distinctive features of *PIGT* loss of function [12]. Microcephaly has its highest prevalence in patients with *PIGK* and *PIGS* involvement, while *PIGT* mutated can be macrocephalic [11,12,14]. Seizures are very common in all the forms described, but they are more frequently controlled by medication in PIGK, GPAA1 and PIGU patients; seizures are often in-tractable, on the other hand, in the case of PIGS or PIGT variants [6,11,12,13,14]. A thin *corpus callosum* has been reported in association with *PIGU* variants [13]; pontine hypoplasia is typical of *PIGS* variants [15].

Transamidase complex disorders related to *GPAA1* defects are very rare. To our knowledge, seventeen patients have been reported at the moment [6,7]. Our patient shares with them the main clinical features, including a severe developmental delay, hypotonia, epilepsy, and nystagmus (Table 1). On the other hand, he does not show osteopenia, as reported in 100% of the patients described by Nguyen [6], and despite what was assessed in other cases, he has a relative macrocephaly. A distinctive feature is the presence of preauricular and cheek skin tags, recalling those observed in Goldenhar syndrome [16].

Among the patients previously described, eleven of them are compound heterozygous for *GPAA1* variants, and six of them are homozygous. Up to now, nineteen variants have been reported: thirteen missenses, five frameshifts, and one located in a splicing site (Table 1). All the individuals carry two missense variants, or one missense and one nonsense. No subject with a double-nonsense variant has been observed. We can hypothesize that a residual activity of the protein is necessary to complete embryonic development, consistent with the lack of animal models with GPI biosynthesis genes null mutants [10].

Our patient carries the homozygous c.424G > A (p.Glu142Lys), a novel missense variant. The homozygosity for very rare variants was favored by consanguinity, albeit of a low degree, between the patient’s parents (two great-great-great grandfathers of the child were brothers). Marriage between consanguineous persons, in turn, may have been favored by a situation of “genetic isolation”, as the two parents come from two very small, neighboring towns in the province of Benevento (Campania, Italy). These two small villages were originally populated by the inhabitants of a castle and are located in an internal, rural area whose history is characterized by poor connections to the region’s larger towns and low migration to them.

The concept of a genetic isolate refers to a population or group of individuals that is relatively isolated from other populations, leading to limited gene flow between them. This isolation can occur due to various factors, such as geographic barriers, cultural practices, or social dynamics. In a genetic isolate, individuals within the population tend to mate and reproduce predominantly within their own group rather than with individuals from other populations. This restricted gene flow can have significant implications for the genetic makeup of the isolated population over time, ultimately favoring the chance of a carrier of rare recessive variants of mating with another carrier of the same variants.

The diagnosis of other affected individuals will allow us to expand the phenotypic and genotypic characterization of this syndrome and assess the long-term prognosis and management of these patients.

## Figures and Tables

**Table 1:** Variants and clinical phenotypes of the reported glycosylphosphatidylinositol biosynthesis defect 15 patients, including this case.

	Variants	Inheritance	Age Years	Height %	Weight %	OFC %	DD ID	Hypotonia	Cerebellar Atrophy	Epilepsy	Nystagmus	Ataxia	Osteo-Penia
This Case	c.424G > A	homoz	4	10	5	55	+	+	+	+	+	+	-
N. 1a	c.872T > C c.981_993del	compound heteroz	15	8	58	14	+	+	+	+	+	+	+
N. 1b	c.872T > C c.981_993del	compound heteroz	10	4	16	27	+	+	+	+	+	+	+
N. 2	c.152C > T c.1164 + 5C > T	compound heteroz	6	59	30	87	+	+	-	+	-	NA	+
N. 3a	c.920delG c.1165G > C	compound heteroz	10	1	49	1	+	+	+	+	+	NA	+
N. 3b	c.920delG c.1165G > C	compound heteroz	3	15	19	19	+	+	+	+	+	NA	+
N. 4a	c.527G > C	homoz	8	1	27	31	+	+	+	-	+	+	+
N. 4b	c.527G > C	homoz	5	1	23	4	+	+	+	-	-	+	+
N. 4c	c.527G > C	homoz	4	1	31	18	+	+	+	-	+	+	+
N. 5a	c.160_161delinsAA c.869T > C	compound heteroz	30	−2 SD	48	50	+	+	+	+	+	+	NA
N. 5b	c.160_161delinsAA c.869T > C	compound heteroz	25	−1.8 SD	31	50	+	+	+	+	+	+	NA
C. I	c.164T > C	homoz	38	17	72	93	+	+	+	+	-	+	+
C. II	c.1049T > G	homoz	1	<1	23	13	+	-	-	-	-	-	NA
C. III	c.917A > G c.1559T > G	compound heteroz	3	<1	3	1	+	+	+	+	-	-	NA
C. IV	c.947C > T	homoz	5	33	96	<1	+	+	-	+	+	+	NA
C. V	c.947C > T c.1233_1239del	compound heteroz	3	1	<1	2	+	+	+	+	+	-	NA
C. VI	c.1477_1478del c.1831T > C	compound heteroz	5	7	31	69	+	+	NA	+	-	-	NA
C. VII	c.619delA c.149T > A	compound heteroz	3	59	72	34	+	+	-	+	-	+	-

Abbreviations. % = percentile, + = present, − = absent, N. = Nguyen and coll. [1], C. = Castle and coll. [5], OFC = occipitofrontal circumference, DD = developmental delay, ID = intellectual disability, NA = not available, SD = standard deviations, homoz. = homozygous, compound eteroz. = compound heterozygous.

## Data Availability

Data available on request due to restrictions.

## Abbreviations

ACMG, American College of Medical Genetics; BP1, Supporting Benign criterion number 1; CADD score, Combined Annotation Dependent Depletion score (a tool for scoring the deleteriousness of single nucleotide variants); GnomAD, The Genome Aggregation Database; *GPAA1*, glycosylphosfatidilinosti anchor attachment protein 1; GPI, glycophosphatidylinositol; MRI, Magnetic resonance imaging; NGS, next-generation sequencing; PIGK, phosphatidylinositol-glycan anchor biosynthesis class K protein; PIGS, phosphatidylinositol-glycan anchor biosynthesis class S protein; PIGT, phosphatidylinositol-glycan anchor biosynthesis class T protein; PIGU, phosphatidylinositol-glycan anchor biosynthesis class U protein; PLPs, likely-pathogenic variants; PM2, Moderate Pathogenic criterion number 2; PP4, Supporting Pathogenic criterion number 4; PP3, Supporting Pathogenic criterion number 3; SDS, standard deviations; SIFT score, Sorting Intolerant from Tolerant score; SNP-microarray, Single Nucleotide Polymorphism microarray; T2, T2 weighted image (one of the basic pulse sequences on MRI); VUS, Variant of Uncertain Significance; WES, whole exome sequencing.
